# Denoising Algorithm for High-Resolution and Large-Range Phase-Sensitive SPR Imaging Based on PFA

**DOI:** 10.3390/s25154641

**Published:** 2025-07-26

**Authors:** Zihang Pu, Xuelin Wang, Wanwan Chen, Zhexian Liu, Peng Wang

**Affiliations:** Department of Precision Instrument, Tsinghua University, Beijing 100084, China

**Keywords:** SPR imaging, polarization filter array, phase-sensitive SPR, denoising algorithm, BM3D

## Abstract

Phase-sensitive surface plasmon resonance (SPR) detection is widely employed in molecular dynamics studies and SPR imaging owing to its real-time capability, high sensitivity, and compatibility with imaging systems. A key research objective is to achieve higher measurement resolution of refractive index under optimal dynamic range conditions. We present an enhanced SPR phase imaging system combining a quad-polarization filter array for phase differential detection with a novel polarization pair, block matching, and 4D filtering (PPBM4D) algorithm to extend the dynamic range and enhance resolution. By extending the BM3D framework, PPBM4D leverages inter-polarization correlations to generate virtual measurements for each channel in the quad-polarization filter, enabling more effective noise suppression through collaborative filtering. The algorithm demonstrates 57% instrumental noise reduction and achieves 1.51 × 10^−6^ RIU resolution (1.333–1.393 RIU range). The system’s algorithm performance is validated through stepwise NaCl solution switching experiments (0.0025–0.08%) and protein interaction assays (0.15625–20 μg/mL). This advancement establishes a robust framework for high-resolution SPR applications across a broad dynamic range, particularly benefiting live-cell imaging and high-throughput screening.

## 1. Introduction

Surface plasmon resonance (SPR) technology has emerged as the gold standard for molecular interaction analysis owing to its capability for rapid, sensitive, and label-free detection [1,2,3]. This powerful analytical technique has found widespread application in protein binding kinetics studies, antibody screening, and various molecular interaction analyses [1,4,5,6]. The sensing principle relies on the sensitivity of the plasmon excitation condition to the refractive index of the surrounding medium [1,2,3]. The main plasmon excitation conditions include the resonance angle, the resonance wavelength, the intensity of the reflected light, and the phase of the reflected light [2,7,8]. Among these, phase-based SPR detection methods have gained significant research attention owing to their superior resolution and straightforward integration into imaging systems [2,9].

Despite these advantages, phase-sensitive SPR detection faces a fundamental challenge: the inverse relationship between the detection range and resolution of refraction index [10,11]. This limitation poses a significant obstacle for studies simultaneously relying on a high resolution and a broad measurement range. Consequently, applications such as cellular SPR imaging, solution differentiation assays, and comprehensive biomolecular interaction studies under different solutions remain constrained by this inherent compromise [11,12,13].

Several approaches for achieving a wide detection range have been reported, including wavelength scanning and angle scanning techniques [10]. Although these methods effectively extend the measurement range for changing the resonance conditions during measurement, they inherently reduce the time resolution because of the scanning process. In addition, angle scanning presents certain challenges for image alignment [14]. Alternative strategies aim at material modifications, such as employing ZnSe-based prisms or utilizing multi-component metal films as substitutes for conventional gold films [11,15]. Research on fiber-based SPR has also boosted improvement of the large detection range [16].

Improving the refractive index resolution is an important focus of SPR technology [17,18,19]. The fundamental challenge in achieving a higher resolution lies in effective noise suppression, where light source fluctuations and sensor detection noise constitute the predominant noise sources [20,21,22]. To suppress the influence of light source fluctuations, some studies have adopted self-referencing approaches, which require additional optical configurations such as masking on the sensor chip [2,23,24,25]. Alternative differential methods, including our previously developed differential interferometric imaging scheme [26] and polarization camera approach [17,27], have been employed to eliminate common-mode noise. Regarding sensor detection noise, as far as we know, most existing solutions rely on data processing techniques, particularly simple temporal smoothing filters [17,28]. However, such filtering approaches inherently compromise temporal resolution, which is a key advantage of SPR technology in capturing rapid molecular binding dynamics. In angle or wavelength scanning methods, neural networks have been applied to denoise scanning data (curve) and extract the exact refractive index (value) [29,30]. However, in phase imaging methods, little extra information can be used to denoise. Consequently, a detection scheme capable of simultaneously achieving both wide dynamic range and high refractive index resolution, coupled with appropriate data processing methods, presents significant scientific appeal and practical value.

In this study, we developed an enhanced phase detection scheme for SPR imaging that simultaneously achieves a wide-range measurement capability and high refractive index resolution. By advancing the dual-differential interference methodology and implementing a four-polarization filter array (PFA) camera system, we created an optical configuration that efficiently captures the phase difference between the p- and s- polarized components of the SPR reflected beam. This configuration realizes a large detection range of the refractive index. Through differential techniques, the optical configuration eliminates the main common-mode noise caused by light source fluctuations.

To further improve resolution, we developed a novel polarization pair, block matching, and 4D filtering (PPBM4D) algorithm based on modified BM3D/BM4D denoising framework [31,32]. This adaptation specifically addresses the characteristics of the four polarization images, particularly the textural similarity and light intensity redundancy across polarization states. By generating virtual independent measurements for each polarization channel through inter-polarization intensity correlations, the algorithm provides additional constraints for collaborative filtering. Therefore, the implemented system achieves a refractive index detection resolution of 1.51 × 10^−6^ RIU within a wide measurement range of 1.333–1.393 RIU. The system’s performance is validated through ultra-dilute NaCl solution switching (0.0025–0.08%) and biomolecular interaction studies, accurately quantifying antibody–protein binding kinetics down to 0.15625 μg/mL, with an equilibrium dissociation constant (KD) of 1.97 × 10^−9^ M, consistent with Biacore 8 K, the commercial SPR scientific instrument. This advancement significantly expands the capabilities of phase-based SPR imaging, enabling high-resolution studies in live cell membrane dynamics, high-throughput multi-condition binding kinetics, and trace molecular detection.

## 2. Principles

### 2.1. Quad-Polarization Filter Array Imaging Optical Configuration

**Figure 1:**
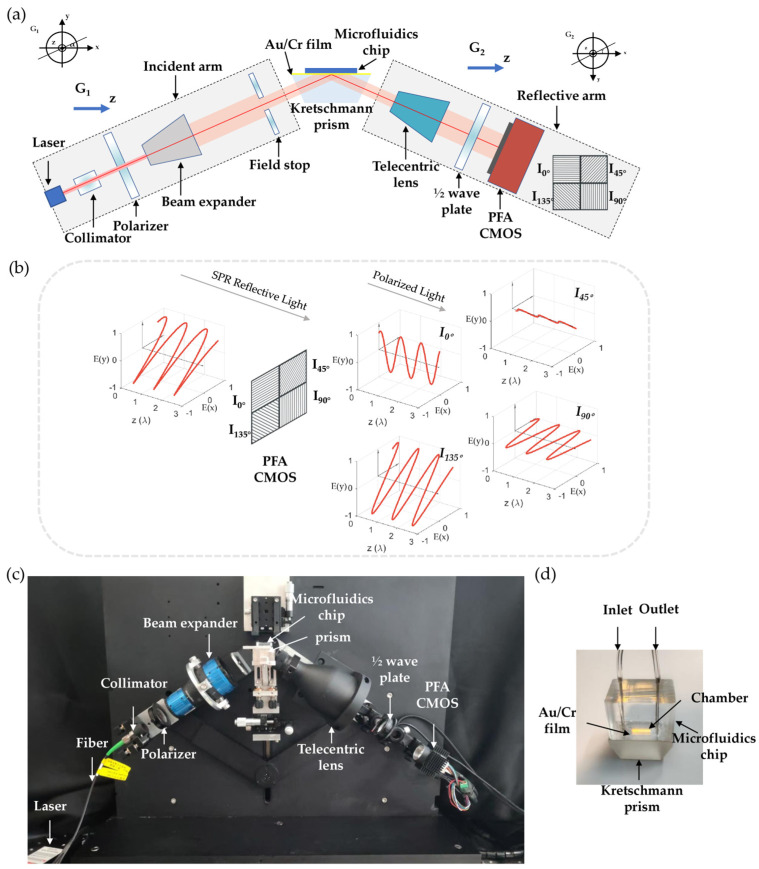
(**a**). Schematic illustration of the optical configuration based on PFA. (**b**). Basic principle of PFA. (**c**). Photograph of the experimental setup. (**d**). Photograph of detailed microfluidics chip and prism structures.

The polarization filter array (PFA) represents a novel imaging device that integrates micro-polarization filter arrays with CMOS sensors, enabling simultaneous acquisition of light intensity, angle of polarization (AoP), and degree of polarization (DoP) information. This technology has found widespread applications in remote sensing, architectural imaging, and defect detecting tasks [33,34]. Recent studies have also demonstrated its potential for SPR imaging applications [17,27]. Building upon these developments, we have designed an improved SPR imaging detection system based on a PFA CMOS, with the corresponding optical schematic configuration and experimental setup shown in Figure 1a. And a brief principle introduction to the PFA camera is shown in Figure 1b for better understanding.

A photograph of the experimental setup is shown in Figure 1c. A 633 nm laser (Changchun New Industries Optoelectronics Technology Co., Ltd., Changchun, China) serves as the light source, with the beam undergoing collimation, polarization, expansion, and spatial filtering through aperture masking (Daheng Optics, Beijing, China). The conditioned beam illuminates a Kretschmann prism (ZF5 glass, *n* = 1.734) coated with 3 nm Cr and 30 nm Au layers for SPR excitation. The reflected light is then magnified through an inverse telecentric lens (Daheng Optics, Beijing, China), phase-modulated by a half-wave plate before being finally captured by a quad-polarization filter array sensor (Sony IMX250 CRZ, Tokyo, Japan) for real-time image acquisition. The acquisition rate is set to 2 Hz, with both images and SPR phase curves being plotted simultaneously on the computer for real-time monitoring. Figure 1d gives a more detailed introduction to the prism and microfluidics chip, and the solution can flow within the chamber above the film. The measuring system features a thermally insulated enclosure to minimize the external temperature influences on SPR signals.

### 2.2. Optical Path Modulation Model

SPR phase detection relies on the characteristic phase shift of reflected light that varies with the dielectric properties of analytes near the gold surface. This phenomenon is well described by the Fresnel multilayer reflection model and wavevector matching conditions at the metal–dielectric interface—established theoretical frameworks in SPR research [35,36]. When SPR occurs, the p-polarized component undergoes both intensity attenuation and phase jump due to energy transfer to surface plasmons, while the s- polarized component remains largely unaffected as a reference. These differentially modulated p- and s- polarized states, now carrying SPR-induced phase and intensity signatures, subsequently enter our quad-polarization optical modulation system for phase extraction.

The Jones calculus method is employed to analyze SPR signals acquired through different polarization channels of the quad-polarization camera. The incident light becomes linearly polarized after passing through the polarizer, with α representing the polarization angle and $E_{i}$ denoting the instantaneous amplitude:

(1) $$E_{i}=E_{i0}\left[\begin{array}{c} cos\alpha \\ sin\alpha \end{array}\right]$$

The Jones matrix for SPR reflection is expressed as follows:

(2) $$J_{SPR}=\left[\begin{array}{cc} \left|r_{s}\right|e^{i\varphi_{s}} & 0\\ 0 & \left|r_{p}\right|e^{i\varphi_{p}} \end{array}\right]$$

where the components can be calculated using the Fresnel equations [34,35]. To enhance intensity contrast among the four polarization pixels and enable effective acquisition of both p- and s- polarized components at 45° and 135° orientations, the fast axis of the half-wave plate is designed at γ = 22.5° relative to the s-polarization direction, with its Jones matrix given by:

(3) $$J_{1/2}=\left[\begin{array}{cc} \sqrt{2}/2 & \sqrt{2}/2\\ \sqrt{2}/2 & -\sqrt{2}/2 \end{array}\right]$$

The Jones matrices for the four polarization filters on the CMOS detector are:

(4) $$J_{\theta }=\left[\begin{array}{cc} \cos^{2}\theta & cos\theta sin\theta \\ cos\theta sin\theta & \sin^{2}\theta \end{array}\right]$$

where θ denotes each pixel’s polarization orientation relative to the x-axis, where θ equals 0°, 45°, 90°, and 135° in PFA, separately.

The light intensities detected by the four polarization channels are derived as follows:

(5) $$I_{0{}^{\circ}}={\left|J_{0{}^{\circ}}J_{1/2}J_{SPR}J_{i}\right|}^{2}=\frac{E_{i}^{2}}{2}\left[{\left|r_{s}\right|}^{2}\cos^{2}\alpha +{\left|r_{p}\right|}^{2}\sin^{2}\alpha -2\left|r_{s}\right|\left|r_{p}\right|\sin\alpha \cos\alpha \cos\left(\varphi_{p}-\varphi_{s}\right)\right]$$

(6) $$I_{90{}^{\circ}}={\left|J_{90{}^{\circ}}J_{1/2}J_{SPR}J_{i}\right|}^{2}=\frac{E_{i}^{2}}{2}\left[{\left|r_{s}\right|}^{2}\cos^{2}\alpha +{\left|r_{p}\right|}^{2}\sin^{2}\alpha +2\left|r_{s}\right|\left|r_{p}\right|\sin\alpha \cos\alpha \cos\left(\varphi_{p}-\varphi_{s}\right)\right]$$

(7) $$I_{45{}^{\circ}}={\left|J_{45{}^{\circ}}J_{1/2}J_{SPR}J_{i}\right|}^{2}=E_{i}^{2}{\left|r_{p}\right|}^{2}\cos^{2}\alpha$$

(8) $$I_{135{}^{\circ}}={\left|J_{135{}^{\circ}}J_{1/2}J_{SPR}J_{i}\right|}^{2}=E_{i}^{2}{\left|r_{s}\right|}^{2}\mathrm{si}n^{2}\alpha$$

Through this innovative design, with the half-wave plate’s fast axis oriented at 22.5°, we achieve complementary interference patterns in $I_{0{}^{\circ}}$ and $I_{90{}^{\circ}}$, while $I_{45{}^{\circ}}$ and $I_{135{}^{\circ}}$ directly represent the p- and s- polarized intensities, respectively. 

This configuration enables the derivation of a key parameter:

(9) $$\begin{array}{cc} \cos\left(\Delta_{\varphi }\right) & =\frac{I_{0{}^{\circ}}-I_{90{}^{\circ}}}{2\sqrt{I_{45{}^{\circ}}I_{135{}^{\circ}}}}\\ & =\frac{\frac{E_{i}^{2}}{2}\left[{\left|r_{s}\right|}^{2}\cos^{2}\alpha +{\left|r_{p}\right|}^{2}\sin^{2}\alpha +2\left|r_{s}\right|\left|r_{p}\right|\sin\alpha \cos\alpha \cos\left(\varphi_{p}-\varphi_{s}\right)\right]}{2\sqrt{E_{i}^{2}{\left|r_{p}\right|}^{2}\cos^{2}\alpha {\times E}_{i}^{2}{\left|r_{s}\right|}^{2}\mathrm{si}n^{2}\alpha }}\\ & -\frac{\frac{E_{i}^{2}}{2}\left[{\left|r_{s}\right|}^{2}\cos^{2}\alpha +{\left|r_{p}\right|}^{2}\sin^{2}\alpha -2\left|r_{s}\right|\left|r_{p}\right|\sin\alpha \cos\alpha \cos\left(\varphi_{p}-\varphi_{s}\right)\right]}{2\sqrt{E_{i}^{2}{\left|r_{p}\right|}^{2}\cos^{2}\alpha {\times E}_{i}^{2}{\left|r_{s}\right|}^{2}\mathrm{si}n^{2}\alpha }}\\ & =\frac{2E_{i}^{2}\left|r_{s}\right|\left|r_{p}\right|\sin\alpha \cos\alpha \cos\left(\varphi_{p}-\varphi_{s}\right)}{2E_{i}^{2}\left|r_{s}\right|\left|r_{p}\right|\sin\alpha \cos\alpha }=\cos\left(\varphi_{p}-\varphi_{s}\right) \end{array}$$

The ${\frac{E_{i}^{2}}{2}\left|r_{s}\right|}^{2}\cos^{2}\alpha +{\frac{E_{i}^{2}}{2}\left|r_{p}\right|}^{2}\sin^{2}\alpha$ part in both $I_{0{}^{\circ}}$ and $I_{90{}^{\circ}}$ is the common-mode interference part of the light in two polarization directions, and this part is subtracted to zero in $I_{0{}^{\circ}}-I_{90{}^{\circ}}$, which shows the common-mode noise suppression of the polarization-based method. $I_{45{}^{\circ}}$ and $I_{135{}^{\circ}}$ in the denominator theoretically eliminate the influence of $E_{i}^{2}$, which often fluctuates with the laser power.

Furthermore, the power of the laser is set properly to make the average of $I_{0{}^{\circ}}$ and $I_{90{}^{\circ}}$, occupy half of the CMOS dynamic range to avoid overexposure and insufficient light and to make full use of the CMOS’s performance and reduce the impact of quantization errors. And the polarizer angle α is set to 45° to maximize the difference between $I_{0{}^{\circ}}$ and $I_{90{}^{\circ}}$, thereby optimizing interference contrast for improved measurement sensitivity. This design ensures optimal performance while maintaining mathematical simplicity in signal processing.

**Figure 2:**
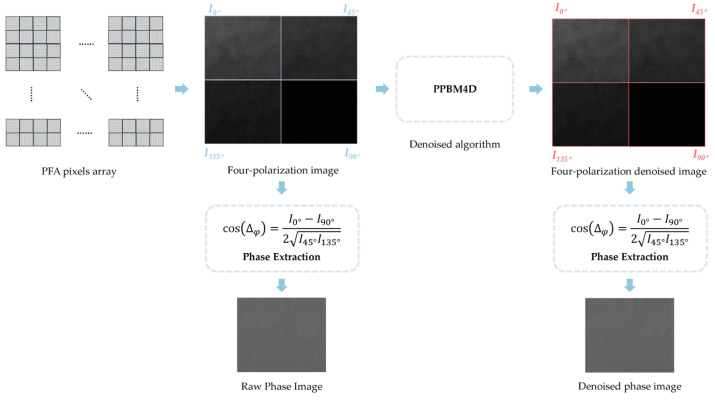
The workflow of the phase image extraction principle.

Figure 2 illustrates the workflow of phase image extraction. The PFA camera captures the initial polarization images where pixels with different polarization orientations are spatially interleaved. In the first processing step, these interleaved pixels are reorganized into separate images based on their polarization directions to facilitate subsequent analysis and observation. The SPR phase image is then obtained using the phase difference calculation formula introduced earlier. Subsequently, the PPBM4D algorithm is applied to denoise the four polarization images. The same phase extraction method is then employed to derive the denoised phase images.

## 3. Materials and Methods

### 3.1. Description of the Denoising Problem

The denoising challenge in SPR imaging primarily stems from two noise sources: global intensity variations caused by light source fluctuations and additive noise components, including dark current noise, readout noise, and shot noise, introduced during image acquisition [20,21,22]. While the differential phase extraction method in PFA effectively eliminates most noise associated with light source variations, the remaining noise can be collectively modeled as additive Gaussian noise in the images, resulting in Gaussian-distributed phase measurement errors [17].

Since the system’s refractive index resolution is conventionally defined as three standard deviations (3σ) of the measurement noise [17,30], there exist two algorithmic approaches to enhance resolution: either denoising the raw detection images prior to phase calculation or processing the time-series phase data directly. Current literature reveals that scanning-based SPR detection systems (wavelength scan or angle scan) have successfully incorporated various advanced data processing techniques, like artificial neural networks (ANN) or deep neural networks (DNN) [21,30]. However, phase imaging methods still predominantly rely on simple smoothing filters [17,28], which, while effective for slowly varying signals, inevitably compromise temporal resolution and dynamic characteristics.

To bridge this gap, we develop PPBM4D, a denoising algorithm that exploits the intrinsic intensity redundancy between orthogonally polarized image pairs in our quad-polarization PFA system. Based on BM3D’s collaborative filtering framework, this optimized approach enhances SPR polarization image denoising while improving refractive index measurement resolution.

### 3.2. Brief Introduction to BM3D/BM4D Algorithms

BM3D represents a powerful collaborative filtering denoising approach that operates through three fundamental steps: (1) block matching to identify similar image patches, (2) 3D transformation and collaborative filtering of grouped patches, and (3) aggregation of filtered results to produce the final denoised output. The method employs a two-stage processing pipeline for enhanced performance. A raw denoising pass creates a basic estimate that serves as a reference for the second stage, where Wiener filtering is applied to further refine the results [31,32].

This method is grounded in rigorous mathematical principles while demonstrating remarkable adaptability, as manifested by its successful extension from grayscale image processing to color image or video sequence denoising. The algorithm’s demonstrated robustness and consistent performance have facilitated its broad adoption across both research and industrial implementations [37]. The algorithm’s flexible framework allows for various extensions while preserving its core denoising performance. This makes it especially suitable for our SPR-specific polarization adaptations.

### 3.3. Brief Introduction to PPBM4D Algorithms

The unique properties of quad-polarization imaging provide critical advantages for enhancing conventional BM3D/BM4D denoising. A fundamental characteristic arises from the fact that light can be decomposed into any pair of orthogonal polarization components [38]. Quad-polarization cameras capture two such orthogonal decompositions: one at 0° and 90°, and another at 45° and 135°. Consequently, the total intensity remains conserved, yielding the identity:

(10) $$I_{0{}^{\circ}}+I_{90{}^{\circ}}=I_{all}=I_{45{}^{\circ}}+I_{135{}^{\circ}}$$

This inherent redundancy in intensity information arises from the independent acquisition by different polarization pixels in a PFA sensor [38]. For instance, while $I_{0{}^{\circ}}$ is directly measured, it can also be virtually derived from the other three polarization components as $I_{0{}^{\circ}}$ = $I_{45{}^{\circ}}$ + $I_{135{}^{\circ}}{-I}_{90{}^{\circ}}$. This redundancy provides additional constraints for denoising algorithms to exploit. Therefore, a polarization pair (PP) generator can be employed in the BM3D/BM4D algorithm, which creates virtual independent measurement.

Furthermore, the block matching process in BM3D/BM4D can be optimized for quad-polarization images by adopting a strategy similar to the color version, block matching, and 3D filtering algorithm’s (C-BM3 D) YUV decomposition [31]. For SPR images, the $I_{0{}^{\circ}}$ component, which contains essential phase information ($\left|r_{s}\right|$, $\left|r_{p}\right|$, $\cos\left(\varphi_{p}-\varphi_{s}\right)$), naturally serves as the optimal reference channel (analogous to the Y channel in color images) for block matching. Thus, the block matching results from $I_{0{}^{\circ}}$ can guide the grouping and denoising of other polarization components. This approach significantly improves computational efficiency compared to conventional single-channel processing.

Building on these insights, we propose the PPBM4D denoising algorithm. The continuous SPR image sequences are treated as video signals. Consistent with the BM3D/BM4D method, the overall algorithm consists of two main steps. The first step is hard-threshold filtering, which includes several sub-steps: generation of polarization image pairs, block matching in $I_{0{}^{\circ}}$, sharing of block matching results, hard-threshold filtering denoising, and aggregation.

First, polarization image pairs are generated using the redundant information from the four polarization states. The generation methods for different polarization directions are expressed in matrix form in equation (11), where the $I_{k}$ means the initial images and ${I^{\prime }}_{k}$ means the generated virtual measurement images. We can call this polarization image pairs generation ‘polarization pair’ generation, which is the PP part in the name of the algorithm.

(11) $$\left[\begin{array}{c} {I^{\prime }}_{0{}^{\circ}}\\ {I^{\prime }}_{45{}^{\circ}}\\ \begin{array}{c} {I^{\prime }}_{90{}^{\circ}}\\ {I^{\prime }}_{135{}^{\circ}} \end{array} \end{array}\right]=\left[\begin{array}{cc} \begin{array}{cc} 0 & 1\\ 1 & 0 \end{array} & \begin{array}{cc} -1 & 1\\ 1 & -1 \end{array}\\ \begin{array}{cc} -1 & 1\\ 1 & -1 \end{array} & \begin{array}{cc} 0 & 1\\ 1 & 0 \end{array} \end{array}\right]\left[\begin{array}{c} I_{0{}^{\circ}}\\ I_{45{}^{\circ}}\\ \begin{array}{c} I_{90{}^{\circ}}\\ I_{135{}^{\circ}} \end{array} \end{array}\right]$$

Next, the generated ${I^{\prime }}_{0{}^{\circ}}$ and initial $I_{0{}^{\circ}}$ are stacked frame by frame for block matching. To avoid temporal abrupt transitions, we incorporate the consecutive frames before each target frame as input for block matching. That is, for each polarization sub-video, the block matching and subsequent denoising algorithm are performed on three-dimensional data composed of four images: $I_{0{}^{\circ}}(t)$, ${I^{\prime }}_{0{}^{\circ}}(t)$, $I_{0{}^{\circ}}(t-1)$, and ${I^{\prime }}_{0{}^{\circ}}(t-1)$. Since $I_{0{}^{\circ}}$ contains the most image information, block matching is performed only on $I_{0{}^{\circ}}$, and the matching results are shared with the other three polarization directions, thereby generating four-dimensional cubes for each polarization sub-video.

The hard-threshold filtering denoising and aggregation step follows, which is implemented similarly to the BM4D method. The four-dimensional image blocks are decomposed into different levels: intra-blocks, inter-blocks, and inter-frames. Specifically, we use 8 × 8 pixel patches with a 40 × 40 search window for block matching. Wavelet transforms and discrete cosine transform are applied to obtain transform coefficients (bior15_2 d wavelet for intra-block, Hadamard for inter-block, and DCT for inter-frame decomposition). The coefficients below the threshold (3 sigma) are then truncated to zero, which typically represent high-frequency noise signals. After hard-threshold filtering, the inverse transforms are applied to reconstruct the four-dimensional blocks, which are then aggregated back into the original three-dimensional video through weighted averaging. 

The second step closely resembles the first, with the same block and search window parameters in the hard-threshold filtering. Block matching is performed again using the video from step one called standard video, with matching results similarly shared based on $I_{0{}^{\circ}}$. Each generated four-dimensional cube undergoes denoising using Wiener filtering with the tuning parameter lambda equal to 1, where the standard video from the first step serves as a guide to preserve image details and reduce low-frequency noise in the original video.

After these two steps, we obtain the denoised four-polarization SPR video, which is then processed further to extract SPR phase and refractive index in the method shown in Figure 2.

**Figure 3:**
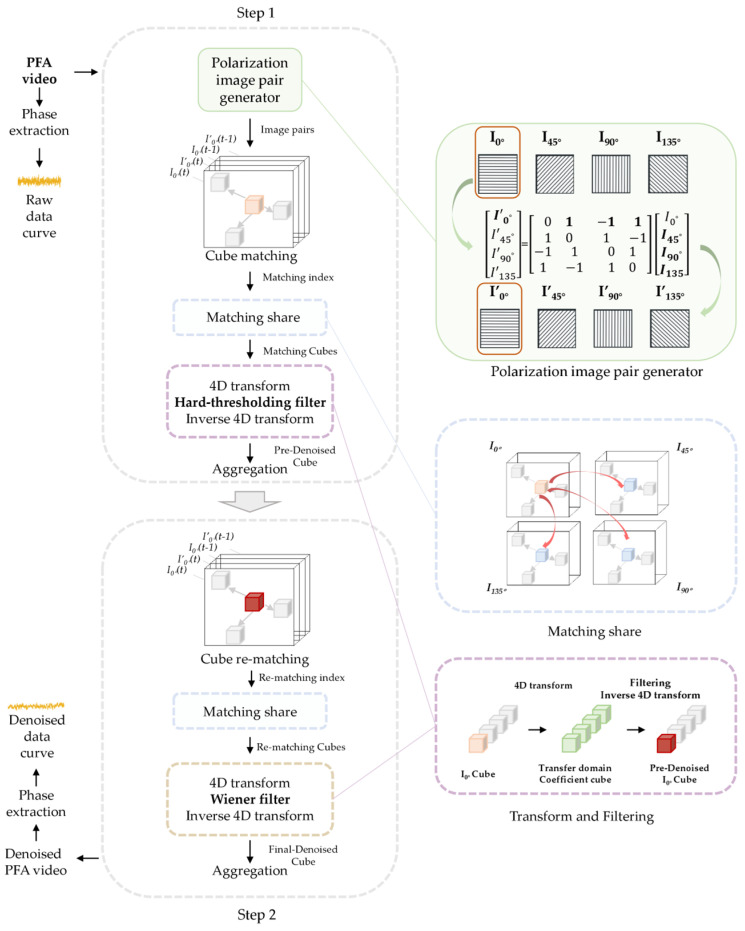
The flowchart of the PPBM4D algorithm.

A flowchart of the entire method is shown in Figure 3. The denoising algorithm is implemented in MATLAB R2022 b (MathWorks, Inc.) and executed on a laptop computer equipped with an AMD Ryzen 7 5800 H processor (3.2 GHz base clock) and 16 GB DDR4 RAM.

### 3.4. Reagents and Experimental Protocols

All reagents were as listed, including 11-mercaptoundecanoic acid (11-MUA), N-hydroxysuccinimide (NHS), and 1-ethyl-3-(3-dimethylaminopropyl) carbodiimide hydrochloride (EDC) from Sigma-Aldrich; rabbit IgG and goat anti-rabbit IgG from HuaYueYang Biotechnology; bovine serum albumin (BSA), phosphate-buffered saline (PBS pH = 7.2–7.4), and glycerol from Solarbio; and glycine buffer solution from Macklin.

**Figure 4:**
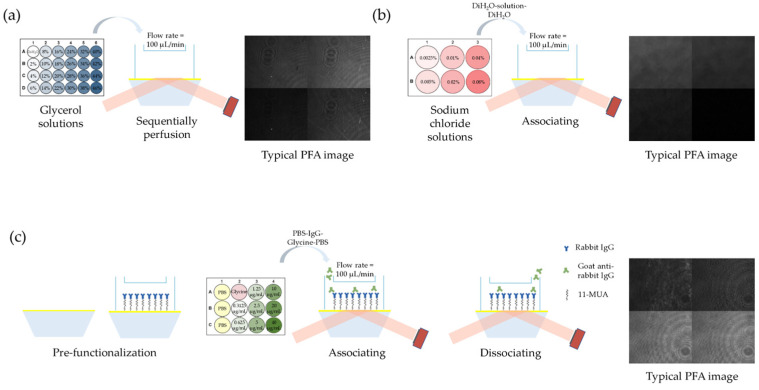
The flowchart of experimental protocols. (**a**). Protocol of glycerol-deionized water solutions switching. (**b**). Protocol of NaCl-deionized water solution switching. (**c**). Protocol of protein binding kinetics measurement.

For system dynamic range and sensitivity calibration, glycerol-deionized water solutions (0–46% mass fraction in 2% increments) were perfused sequentially at 100 μL/min after establishing a deionized water baseline, with each solution maintained for about 5 min. SPR images were recorded to extract phase information and calibrate the refractive index-dependent response. Resolution verification employed NaCl-deionized water solutions (0.08%, 0.04%, 0.25%, 0.01%, 0.005%, and 0.0025%) at 100 μL/min for about 3 min, followed by reversion to deionized water to confirm baseline recovery. Figure 4a and Figure 4b show these protocols, separately.

As for the protein binding kinetics measurement protocol, the sensor chip was prepared through sequential surface functionalization, as shown in Figure 4c. The gold film was first modified via self-assembly in 11-MUA ethanol solution, followed by chemical activation using an equal-volume mixture of 100 mM NHS and 400 mM EDC. Antibodies (rabbit IgG) were immobilized from a 10 mM sodium acetate buffer (pH 5.5), after which the surface was passivated with BSA to block residual reactive groups. During measurements, a stable PBS baseline (100 μL/min, 5 min) preceded injections of goat anti-rabbit IgG (20, 10, 5, 2.5, 1.25, 0.625, 0.3125, and 0.15625 μg/mL). Each binding phase (about 3 min) was followed by dissociation monitoring under PBS flow (10 min) and regeneration with glycine buffer solution (pH = 2.0, 1 min).

Since SPR is inherently sensitive to environmental factors such as temperature [39,40], maintaining stable ambient conditions is crucial. All experiments were conducted in a temperature-controlled laboratory environment with regulated air conditioning and the measuring system featured a thermally insulated enclosure. All reagents and sensor chips stored under 4 degree centigrade were pre-equilibrated to room temperature prior to use. Additionally, the microfluidics chip, as shown in Figure 1d, was designed with a thermally insulated thick-walled architecture to minimize temperature-induced measurement artifacts.

## 4. Results and Discussion

### 4.1. Measurement Range and Sensitivity Calibration

**Figure 5:**
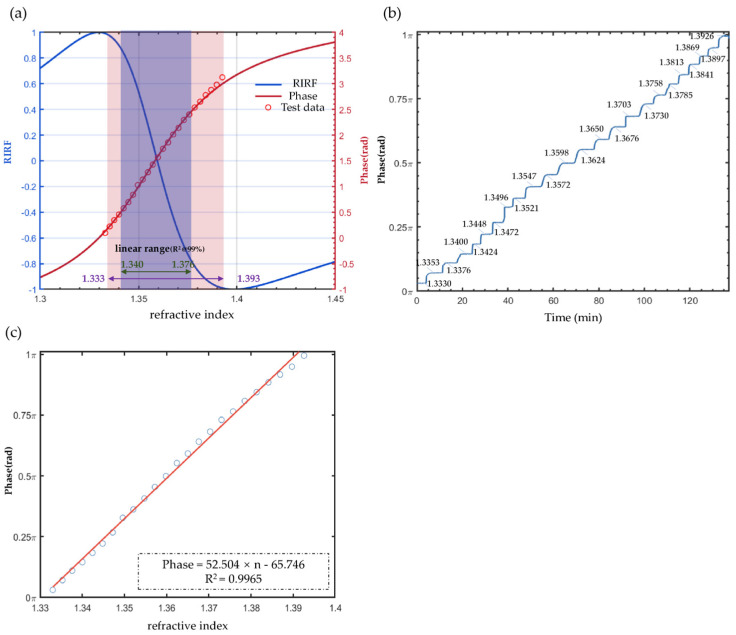
Linear range and sensitivity calibration of the measurement system. (**a**). Theoretical curve of phase and RIRF responses versus refractive index. Blue curve (left axis) displays the RIRF theoretical response with a linear range (R^2^ @99%) of 1.340–1.376, while the red curve (right axis) shows the phase response with an extended linear range (R^2^ @99%) of 1.333–1.393 RIU. The experimental data (red circles) exhibit excellent agreement with the theoretical phase curve. (**b**). Time-resolved phase response during glycerol concentration gradient testing, showing characteristic step-like transitions. The refractive index is marked besides the curve. (**c**). Linear regression of averaged phase values versus glycerol concentrations yields a system sensitivity of 52.504 rad/RIU (R^2^ = 0.9965).

The measurement range and sensitivity of the system were calibrated using glycerol solutions with stepwise concentration gradients. Figure 5a presents the theoretical simulation curve (red) of the PFA detection scheme along with the corresponding experimental results (red circles), demonstrating excellent agreement with the simulated curve. For comparison, we also show the simulation refractive index related factor (RIRF) curve (blue) of our previously developed dual-differential interference phase calculation method based on two CCDs and a polarization beam splitter [26]. Under the same standard of 99.5% coefficient of determination R-square, the linear measurement range is extended from 0.036 RIU (1.340–1.376) to 0.06 RIU (1.333–1.393).

Figure 5b,c show the raw SPR phase–time curves obtained during the glycerol concentration gradient tests, as well as the extracted phase-refractive index data points, fitted curves, and fitting results for stepwise concentration gradients. The sensitivity of the system is determined to be 52.504 rad/RIU (R^2^ = 0.9965).

### 4.2. Enhancement of Refractive Index Resolution via PPBM4D

**Figure 6:**
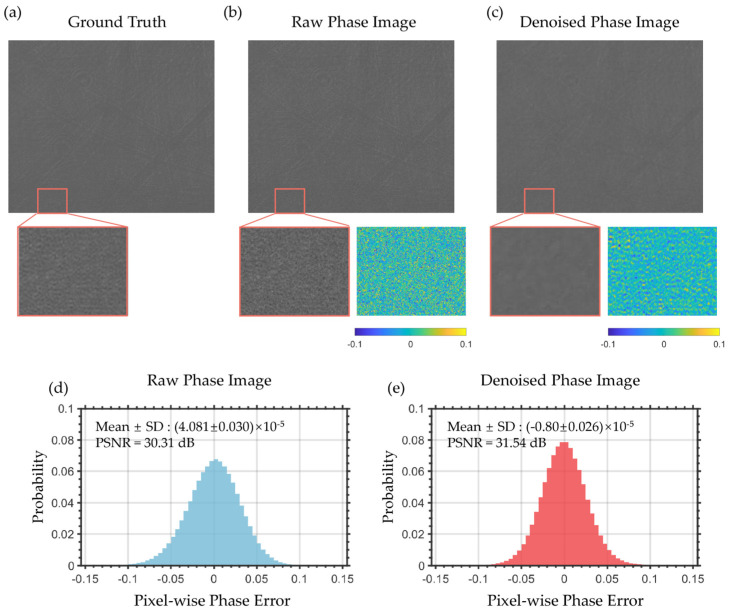
Spatial denoising effect of phase images. (**a**) Ground truth phase image. (**b**) Typical raw phase image. Detailed regions are shown below with pseudo-color difference maps relative to ground truth. (**c**) Denoised phase image. Detailed regions are shown below with pseudo-color difference maps relative to ground truth. (**d**) Pixel-wise phase error histogram of raw single-frame measurement. (**e**) Pixel-wise phase error histogram of denoised single-frame measurement.

We systematically evaluated the denoising performance of the PPBM4D algorithm on SPR phase images in both spatial and temporal domains. For spatial domain analysis, Figure 6 presents experimental results obtained from steady-state deionized water measurements. Figure 6a displays the ground truth (GT) phase image generated by averaging 60 consecutive frames, with an enlarged region of interest (ROI) for detailed comparison. Figure 6b shows a single-frame phase image with corresponding ROI magnification and its difference map with GT in the pseudo-color image, where the difference primarily represents noise components. The denoised result using our algorithm is presented in Figure 6c, along with its magnified view and difference map. The pseudo-color difference maps demonstrate reduced extreme noise values, evidenced by fewer yellow and blue pixels.

We further analyzed the pixel-wise noise distributions for both raw and denoised images in Figure 6d,e. The pixel-wise histograms show that the noise follows a Gaussian distribution in both cases, with the denoised image exhibiting a reduction in both mean noise amplitude and distribution standard deviation. Additionally, the peak signal-to-noise ratio (PSNR) improvement of 1.23 dB further confirms the effectiveness of our PPBM4D method for single-frame denoising.

The core idea of PPBM4D lies in leveraging the noise characteristics from adjacent frames for effective denoising. From an instrumentation perspective, the suppression of temporal phase fluctuations is particularly crucial for improving measurement resolution which is validated through dilute solution switching test below. 

**Figure 7:**
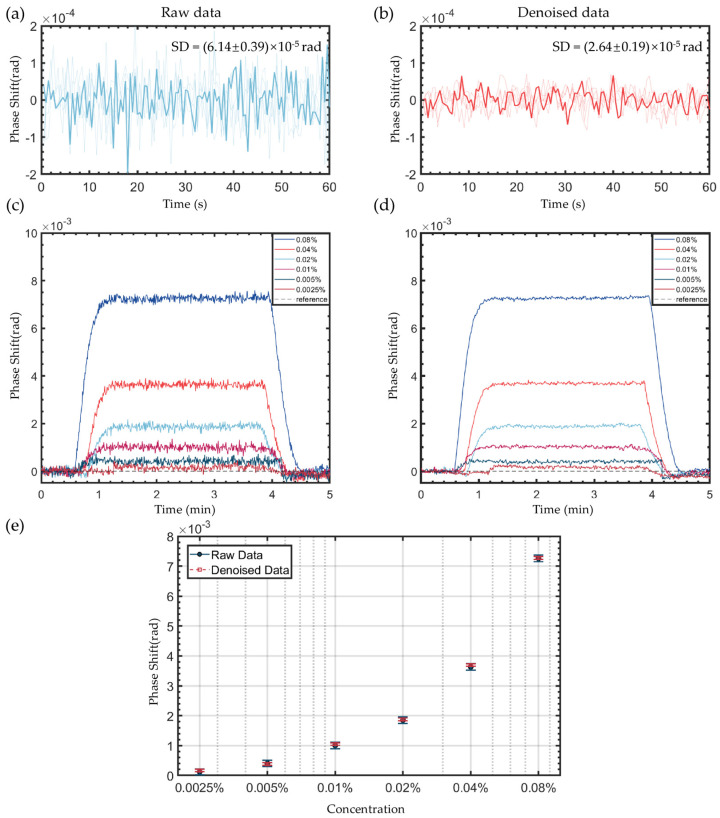
Resolution enhancement validation through dilute solution and noise analysis. (**a**) Raw SPR phase signal in deionized water. The solid line represents the typical curve, while the light-colored lines show results from repeated experiments. (**b**) Denoised phase data in deionized water. The solid line represents the typical curve, while the light-colored lines show results from repeated experiments. (**c**) Raw SPR phase signal in dilute solution of different concentrations. (**d**) Denoised phase data in dilute solution of different concentrations. (**e**) Phase error comparison between raw and denoised data across different concentrations.

Under controlled deionized water flow (100 μL/min), the algorithm reduces the phase noise standard deviation by 57% (from 6.14 × 10^−5^ rad to 2.64 × 10^−5^ rad), while improving the refractive index resolution from 3.51 × 10^−6^ RIU to 1.51 × 10^−6^ RIU, as shown in Figure 7a,b. And Figure 7c,d show the SPR phase curves of different concentrations of NaCl-deionized water solution. Fluctuations are reduced, and as low as 0.0025% NaCl solution (ΔRI ≈ 4 × 10^−6^ RIU) can be easily detected. Figure 7e shows the statistical analysis of the mean phase and error bar of the stable part of the phase curve for different concentrations. The mean data of the raw data and denoised data fit well and the deviations are reduced using the PPBM4D method.

A key innovation of our approach lies in augmenting the BM3D/BM4D framework with a polarization image pair generation mechanism. Comparative analysis was conducted between conventional BM3D/BM4D methods (without polarization image pair generation and our proposed PPBM4D method). As shown in Table 1, while the standard BM4D method demonstrates certain noise reduction capability compared to the raw data, its performance remains inferior to our proposed method. Notably, the BM3D method even increases the noise level in some cases. This phenomenon occurs because BM3D processes individual frames independently without considering inter-frame fluctuations, leading to suboptimal denoising performance. By contrast, our PPBM4D method effectively leverages both spatial and temporal information through the polarization image pairs, resulting in superior noise suppression.

To further optimize the PPBM4D method, we systematically investigated the influence of frame number used in the denoising algorithm on performance. As demonstrated in Table 2, the phase standard deviation decreases with additional frames. Optimal denoising performance is achieved when utilizing either 2 or 3 frames. However, since computational load scales linearly with frame number, we conducted a comprehensive trade-off analysis between processing efficiency and noise suppression effectiveness. Based on this evaluation, the algorithm was ultimately configured to incorporate 2 frames, representing the optimal balance between computational practicality and denoising performance.

Notably, for applications with less temporal resolution requirements, additional smoothing filters can be integrated with our algorithm. For instance, at 0.5 second temporal resolution (5-frame averaging at 10 fps acquisition rate), noise standard deviation improved from (6.14 ± 0.39) × 10^−5^ rad (raw) to (2.36 ± 0.36) ×10^−5^ rad (smoothing only) and further to (1.51 ± 0.21) × 10^−5^ rad (smoothing combined with PPBM4D), corresponding to refractive index resolution enhancements from (1.35 ± 0.21) × 10^−6^ RIU (smoothing only) to (8.63 ± 0.12) × 10^−7^ RIU (smoothing combined with PPBM4D), respectively. Combined with the proposed algorithm and smoothing filter, this scheme provides flexible solutions for high-resolution applications.

### 4.3. Validation of Protein Binding Kinetics Measurement

The determination of kinetic parameters for protein antigen–antibody interactions represents one of the most popular applications of SPR systems. Using our developed system and denoising algorithm, we measured the associating and dissociation processes between goat anti-rabbit IgG and rabbit IgG at varying concentrations, followed by fitting of the equilibrium association constant (KD). The association and dissociation functions are shown as follows:

(12) $$\Gamma_{assoc}\left(t\right)=\Gamma_{max}\times \frac{C_{bulk}}{C_{bulk}+\left(\frac{k_{d}}{k_{a}}\right)}\times \left(1-e^{\left(-t\right)\times \left(k_{a}\times C_{bulk}+k_{d}\right)}\right)$$

(13) $$\Gamma_{dissoc}\left(t\right)=\Gamma_{assoc}\left(t_{PBS}\right)\times e^{-\left(t-t_{PBS}\right)\times k_{d}}$$

where $\Gamma_{assoc}\left(t\right)$ and $\Gamma_{dissoc}\left(t\right)$ describes the association and dissociation phases, respectively. $C_{bulk}$ is the concentration of target IgG solution, $k_{a}$ is the association rate constant, and $k_{d}$ is the dissociation rate constant. KD equals $k_{d}$/$k_{a}$. $\Gamma_{max}$ is the maximal interaction constant, and $t_{PBS}$ is the time moment that PBS solution is injected to start the dissociation phase [41].

**Figure 8:**
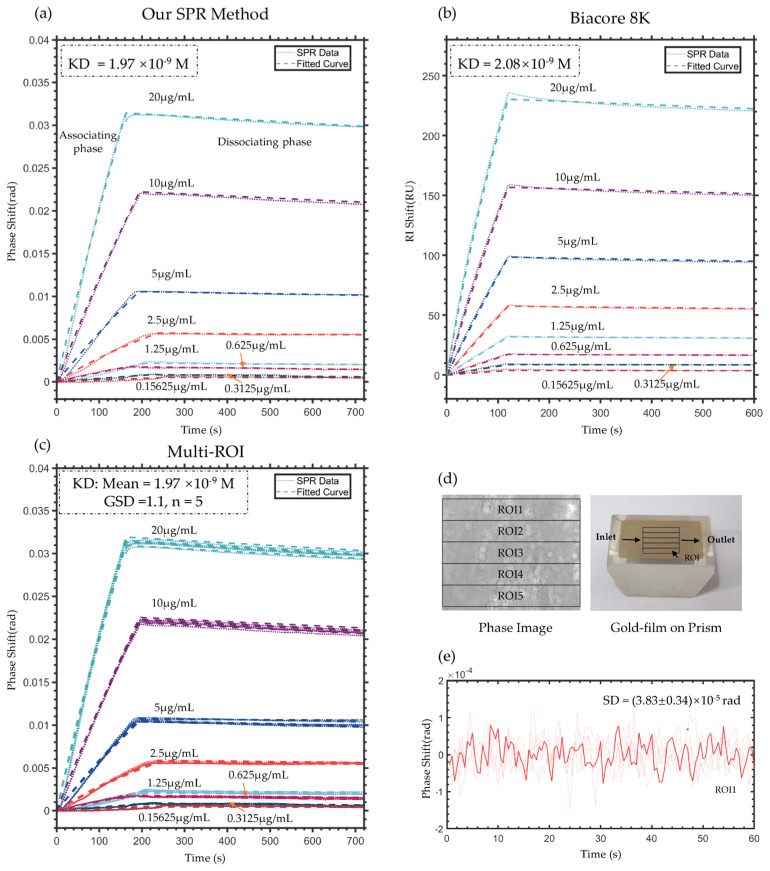
Protein antigen–antibody interaction curves between goat anti-rabbit IgG and rabbit IgG. (**a**) Denoised SPR curves and the fitted curves of the whole ROI in the proposed SPR system and denoised frame. (**b**) Associating and dissociating curves measured by Biacore 8 K. (**c**) Denoised SPR curves and the fitted curves of each ROI in the proposed SPR system and denoised frame. (**d**) ROIs are shown in the phase image and prism. (**e**) Denoised SPR phase signals of a single ROI across multiple tests in deionized water. The solid line represents the typical curve, while the light-colored lines show results from repeated experiments.

The results are presented in Figure 8a. The excellent agreement between fitted curves and experimental data demonstrates the system’s capability to resolve association/dissociation processes at concentrations as low as 0.15625 μg/mL (about 1 nM). The measured equilibrium dissociation constant (KD = 1.97 × 10^−9^ M) shows excellent consistency with the value obtained using Biacore 8 K (KD = 2.08 × 10^−9^ M), the widely-adopted SPR platform in protein interaction studies [42,43], with only a 5.3% relative difference.

To better show the advantage of SPR images, we further investigated the multi-ROI SPR curve and fitted KD in high-throughput applications. The results in Figure 8c indicate that the KD of different ROIs is similar, with the same mean value and geometric standard deviation (GSD) of 1.1, compared to the results of the whole ROI shown in Figure 8a. The ROIs are shown in Figure 8d in the denoised phase image, and the prism is also given to introduce the direction of the protein flow. The ROI is parallel to the flow direction to reduce the effect of mass transfer. Figure 8e shows the denoised SPR phase signal in deionized water of single ROI1 for example. The standard deviation of phase is 3.83 × 10^−5^ rad and the corresponding resolution of refractive index is 2.19 × 10^−6^ RIU.

These protein kinetic experiments validate that the quad-polarization phase detection system combined with the PPBM4D processing algorithm is fully compatible with conventional SPR detection tasks. Notably, this SPR imaging method combines a wide dynamic range with high RI resolution while additionally providing spatially resolved information. Such capabilities suggest promising potential for advanced applications, including high-throughput molecular binding assays and cellular imaging.

### 4.4. Discussion on Application Limitations and Future Work

Recent advances in refractive index sensing have diversified detection approaches for various biomedical needs. Toma et al. demonstrated a dielectric grating sensor utilizing photonic crystal defect engineering to achieve box-shaped resonance spectra, offering exceptional signal-to-noise ratios for trace protein detection [44]. Meanwhile, Torrijos-Morán et al. developed a dual-mode subwavelength grating waveguide sensor with 1350 nm/RIU bulk sensitivity, optimized for continuous fluid monitoring [45]. For miniaturized systems, Aydin et al. comprehensively reviewed over 400 fiber interferometric sensors, highlighting designs where evanescent field overlap parameters critically determine resolution limits [46]. These innovations provide tailored solutions for scenarios requiring metal-free operation, stable single-wavelength readout, or compact fluidic integration.

In specialized application scenarios, particularly those requiring high-throughput detection and imaging, SPR methods offer unique advantages. As the fundamental structural and functional units of organisms, cells interact with the micro-environment primarily through cell membranes. From a biological research perspective, numerous physiological processes—including signal transduction, transmembrane transport, intracellular responses, and immune recognition—are regulated by the binding of membrane proteins with molecules [47]. From a therapeutic standpoint, the mechanisms of numerous drugs rely on their binding to membrane protein targets. Therefore, studying the interactions between membrane proteins and ligands under live cell conditions holds significant importance for cancer research, immune system studies, and investigations of inflammatory responses.

Investigating membrane protein–ligand interactions with SPR represents a major research focus [6,48]. However, cellular studies present unique challenges for SPR imaging due to the small size of cells, substantial refractive index heterogeneity, and weak detection signals. These challenges demand SPR imaging systems with high spatial resolution, a wide refractive index measurement range, and superior refractive index resolution. The optical design and algorithmic system presented in this work, which achieve both a broad measurement range and enhanced refractive index resolution, provide an effective methodology and technical framework for SPR-based analysis of complex biological samples like cells. Furthermore, the PFA imaging system can be coupled with high-spatial-resolution imaging modalities, which will be the focus of our future research efforts.

Nevertheless, it is important to acknowledge certain inherent limitations when employing SPR for biological reaction detection. Primarily, SPR measurements exhibit relatively weak long-term stabilty due to their high sensitivity to environmental temperature fluctuations, laser wavelength drift, and light intensity variations, all of which may compromise phase signal reliability. Although we implemented temperature control methods, further optimization of thermal stability remains necessary for prolonged dynamic monitoring applications.

Furthermore, SPR technology faces additional challenges when applied to complex biofluids (e.g., serum, urine, or blood), where nonspecific adsorption from matrix components may interfere with target molecule detection [49,50,51]. While surface modifications such as BSA blocking can partially mitigate these effects, future research should focus on developing more robust antifouling strategies or microfluidic sample processing methods to improve detection reliability in such complex matrices.

## 5. Conclusions

SPR phase imaging represents a crucial detection modality in SPR technology. This study presents an innovative SPR phase detection scheme capable of simultaneously achieving both a wide refractive index range and high resolution. By integrating an enhanced differential interference method with a quad-polarization camera system, we successfully extract phase differences between the p- and s- polarized components of SPR reflected light from four polarization images, effectively eliminating common-mode noise induced by light source fluctuations. Furthermore, building upon the original BM3D denoising algorithm and leveraging the unique characteristics of quad-polarization images, we developed the PPBM4D algorithm specifically for denoising SPR phase images.

The implemented system achieves a refractive index resolution of 1.51 × 10^−6^ RIU within an extensive measurement range of 1.333–1.393 RIU. Experimental validation through ultra-low concentration solution switching and antibody–protein binding measurements confirms both the system’s detection capability and the efficacy of our denoising approach. The measurement resolution could be further enhanced by implementing wavelength-stabilized lasers and improving thermal control systems on the detection chip. The combined quad-polarization acquisition and advanced denoising algorithm establishes a systematic solution for SPR instrumentation with high resolution, while simultaneously providing a versatile platform for complex biological studies such as cellular membrane dynamics investigations and high-throughput multi-channel analysis. This integrated approach bridges optical innovations with computational imaging advancements for next-generation SPR biosensing applications.

## Figures and Tables

**Table 1:** Method comparison.

Method	Standard Deviation (SD) of Phase (Rad)	Resolution of Refractive Index (RIU)
Raw data	(6.14 ± 0.39) ×10^−5^	(3.51 ± 0.22) × 10^−6^
BM3D	(6.54 ± 0.09) ×10^−5^	(3.73 ± 0.05) × 10^−6^
BM4D	(4.33 ± 0.18) × 10^−5^	(2.47 ± 0.10) × 10^−6^
**Our method**	**(2.64 ± 0.19) × 10^−5^**	**(1.51 ± 0.11) × 10^−6^**

**Table 2:** Parameter comparison.

Method	Number of Frame	SD of Phase (Rad)	Resolution of Refractive Index (RIU)
PPBM4D	1	(4.66 ± 0.23) × 10^−5^	(2.66 ± 0.13) × 10^−6^
**PPBM4D (our method)**	**2**	**(2.64 ± 0.19) × 10^−5^**	**(1.51 ± 0.11) × 10^−6^**
PPBM4D	3	(2.63 ± 0.13) × 10^−5^	(1.51 ± 0.07) × 10^−6^

## Data Availability

Data are contained within the article.
